# Factors associated with oral frailty in older adults: a systematic review and meta-analysis

**DOI:** 10.3389/fpubh.2025.1688322

**Published:** 2025-12-10

**Authors:** Yanru Chen, Ling Zhang, Wen Yan, Fan Liu

**Affiliations:** 1 Department of Cardiology and Endodontics, State Key Laboratory of Oral Diseases and National Clinical Research Center for Oral Diseases, West China Hospital of Stomatology, Sichuan University, Chengdu, Sichuan, China; 2 Department of Prosthodontics, State Key Laboratory of Oral Diseases and National Clinical Research Center for Oral Diseases, West China Hospital of Stomatology, Sichuan University, Chengdu, Sichuan, China; 3 Department of Nursing, State Key Laboratory of Oral Diseases and National Clinical Research Center for Oral Diseases, West China Hospital of Stomatology, Sichuan University, Chengdu, Sichuan, China

**Keywords:** oral frailty, older adults, influencing factors, systematic review, meta-analysis

## Abstract

**Background:**

Oral frailty (OF) is defined as a series of age-related declines in oral function that cause malnutrition and many other negative health outcomes. This study aimed to comprehensively examine the factors associated with OF in older adults in order to provide further direction for the early identification and tailored interventions of in this population.

**Methods:**

PubMed, Cochrane Library, Embase, Web of Science, China National Knowledge Infrastructure (CNKI), Chinese Biomedical Database (CBM), CQVIP, and Wanfang databases were comprehensively searched for observational studies from inception to April 2025. All analyses were performed using RevMan5 (version 5.4) and Stata 16.0 software.

**Results:**

A total of 39 studies were selected from 10,863 references, involving 41,854 older adults, and were included in this systematic review. A meta-analysis was performed on the 27 influencing factors. Age (WMD = 4.21, 95% CI: 3.09–5.33), female sex (OR = 1.76, 95% CI: 1.41–2.20), years of education (WMD = 0.68, 95% CI: −0.04-1.41), low income (OR = 1.94, 95% CI: 1.20–2.68), BMI (WMD = 1.50, 95% CI: 0.41–5.59), sarcopenia (OR = 2.92, 95% CI: 2.54–3.36), stroke (OR = 2.68, 95% CI: 1.28–5.61), osteoporosis (OR = 1.50, 95% CI: 1.05–2.13), cognitive impairment (OR = 4.25, 95% CI: 1.72–10.51), ≥2 chronic conditions (OR = 6.07, 95% CI: 2.32–15.90), taking ≥ 5 medications (OR = 2.48, 95% CI: 1.59–3.88), poor sleep quality (OR = 3.79, 95% CI: 1.47–9.80), physical frailty (OR = 2.21, 95% CI: 1.63–3.00), low physical activity level (OR = 1.77, 95% CI: 1.41–2.22), ADL impairment (OR = 4.46, 95% CI: 1.81–11.01), depression (OR = 3.76, 95% CI: 2.69–5.24), social isolation (OR = 1.95, 95% CI: 1.43–2.65), malnutrition (OR = 2.58, 95% CI: 1.55–4.28), low dietary variety (OR = 1.69, 95% CI: 1.37–2.08) and poor appetite (OR = 2.11, 95% CI: 1.62–2.74) were found a significant associations with oral frailty in older adults.

**Conclusion:**

We identified 20 factors associated with oral frailty in older adults, categorized into five primary domains: sociodemographic factors, comorbidity factors, physical function factors, social and psychological factors, and dietary factors. Public health policymakers and healthcare practitioners should undertake a comprehensive evaluation of these associated factors and develop targeted intervention strategies designed to reduce oral frailty, thereby enhancing the overall health of the older population.

**Systematic review registration:**

Identifier [CRD42024588941].

## Introduction

Population aging has become one of the global social issues. In 2022, the world’s older population aged ≥65 years accounted for 9.7% of the total population and is expected to account for 16.4% of the total population by 2050 (1, 2). The United Nations (UN) General Assembly declared the period from 2021 to 2030 as the UN Decade of Healthy Ageing and asked the World Health Organization (WHO) to lead the implementation. It is emphasized that policymakers around the world should focus on improving the lives of older adults (1). Oral health, as an important indicator of human health, is closely related to the overall health and social wellbeing of older adults (3), and the maintenance of oral health is an important means of preventing and controlling systemic diseases (4). However, with global population aging, the oral health of the geriatric population is a matter of significant concern. Oral problems, such as tooth loss, dental caries, periodontal disease, and dry mouth, seriously affect the quality of life of older adults (3).

In order to advance the timing for the prevention and treatment of oral diseases in older adults, the concept of oral frailty (OF) was introduced as a novel concept within the field of frailty in 2013, which was defined as accumulated impairments in multiple oral health aspects and functions (3). These oral function impairments significantly heighten the risk of physical frailty, sarcopenia, malnutrition, and disability in older adults (5–7). A recent meta-analysis focused on the prevalence of oral frailty indicates that the overall prevalence of oral frailty among older adults is 32% (95% CI, 24–40%). However, the data exhibit significant heterogeneity driven by factors such as country, measurement method, and publication year (8, 9). It is evidenced by previous research that the syndrome is reversible in the early stages of, and if intervention measures are actively applied, further deterioration can be prevented (5, 10). Therefore, the identification of factors associated with OF is crucial for the early screening of high-risk populations and the implementation of effective interventions and health management strategies, thereby reducing OF in older adults and the adverse consequences associated with it.

Recently, a growing number of studies have focused on the current status of and their associated factors as researchers and health managers have gained a better understanding of the condition. These studies indicate that factors such as age, education level, physical weakness, cognitive decline, depression, diabetes, nutritional status, and polypharmacy all influence OF in older adults. However, because of variations in study populations, geographical locations, and assessment methods, along with a lack of large-scale studies, the findings can differ significantly or even contradict each other. This highlights the need for a synthesis of available evidence to draw more reliable conclusions. Hence, this study aimed to thoroughly investigate the factors associated with OF in older adults through a systematic review and meta-analysis in order to provide further direction for the early identification and tailored interventions of in older adults, aiming to decrease the incidence of and its negative effects, ultimately enhancing their long-term quality of life.

## Methods

We published a systematic review protocol on PROSPERO (registration No. CRD42024588941) and performed the review according to the Preferred Reporting Items for Systematic Review and Meta-Analysis (PRISMA) standards. Data supporting the findings of this study are available from the corresponding author upon request.

### Literature search strategy

We searched PubMed, Web of Science, Cochrane Library, Embase databases, China National Knowledge Infrastructure (CNKI), Wanfang Database, CQVIP, and Chinese Biomedical Literature (CBM) from inception to April 2025. The literature was limited to English and Chinese. The search strategies were performed using a combination of MeSH terms and free words. Additionally, the reference lists included in the identified articles were manually searched to identify additional relevant publications. This study did not require the approval of an ethics committee, as it relied solely on previously published studies. Specific search strategies are listed in Supplementary Table S1.

### Inclusion and exclusion criteria

Two researchers (Chen and Zhang) conducted the literature search and extracted the data independently. Discrepancies were resolved by a third researcher (Yan). The inclusion criteria of a study in the systematic review were as follows: (1) case–control, cohort, or cross-sectional studies; (2) study subjects were older adults (≥60 years old), regardless of race or nationality; (3) there was a clear definition and evaluation method for OF; and (4) the outcome indicators were the factors associated with OF in older adults. The exclusion criteria were as follows: (1) reviews, conference proceedings, case reports, comments, and abstracts; (2) studies for which the full text was unavailable or data could not be extracted; (3) low-quality literature (AHRQ score <4 or NOS score <6); and (4) the language of the publication was other than English or Chinese.

### Data extraction

Key information from the included studies was extracted by two researchers, working independently, using a data extraction form. From each study, we extracted the following information: first author’s name, publication year, country, study design, sample source, mean age (y), sex (m/f), assessment tool, prevalence, and associated factors (Table 1). All extracted data were stored in Microsoft Excel.

**Table 1:** Characteristics of included studies (*n* = 39).

Author, year	Country	Study Design	Sample source	Mean age (y)	Sample (M/F)	Assessment tool	Prevalence	Associated factors	NOs/ AHRQ score
Feng, 2024 (17)	China	①	Community	71.26 ± 7.33	427/411	OFI-8 score	29.24%	Age, primary and lower education level, living alone, no spouse, monthly incomes <3,000 CNY, smoking, drinking alcohol, diabetes, xerostomia, ≥4 medications, poor sleep quality, malnutrition, physical frailty, periodic physical examination	7
Hu, 2024 (5)	China	①	Community	74.98 ± 6.04	209/171	OFI-8 score	/	Age, gender, education level, physical frailty, number of dentures, xerostomia, subjective chewing difficulty, oral health score, sleep quality	7
Jiang, 2025 (18)	China	①	Community	69.86 ± 7.89	1604/1459	OFI-8 score	46.82%	Age, disability, sarcopenia, social isolation, subjective cognitive decline	5
Jiao, 2023 (20)	China	①	Long-term care facilities	79.49 ± 10.50	139/131	OFI-8 score	25. 19%	Age, ≥2 chronic conditions, drinking alcohol, ADL impairment, malnutrition	9
Lin, 2022 (25)	China	①	Community	≥65	314/786	OFI-6 score	20.70%	≥75 years old, late-life depression	9
Luo, 2025 (13)	China	①	Hospital	71.44 ± 7.47	197/234	OFI-8 score	32.95%	Age, education, dysphagia, HbA1c, OHAT, remaining teeth, GSEOH	9
Ma, 2025 (14)	China	①	Hospital	≥60	252/199	OFI-8 score	47.67%	Age, physical frailty, ≥2 chronic conditions, NRS 2002, NIHSS, GOHAI, BI	9
Sun, 2025 (15)	China	①	Communities, hospitals, parks, streets, and fields.	≥60	662/791	OFI-8 score	51.41%	Depressive symptoms	8
Tang, 2023 (21)	China	①	Community	72.70 ± 6.30	536/762	OFI-8 score	44.68%	Gender, age, sleep quality, chronic diseases, depressive symptoms, a diet mainly of meat dishes, low levels of social support, and poor salty taste	7
Tu, 2023 (22)	China	①	Community	72.71 ± 8.00	92/112	OFI-8 score	33.82%	Age, gender, primary and lower education level, polypharmacy, physical frailty, number of dentures, xerostomia, subjective chewing difficulties, oral health score	7
Wang, 2023 (23)	China	①	Community	≥60	103/120	OFI-8 score	59.19%	≥80 years old, monthly income < 3,000 CNY, smoking, oral health-related self-efficacy	7
Wang, 2024 (16)	China	①	Community	60–92	209/269	OFI-8 score	71.55%	Gender, age, nation, personal monthly income, drinking, number of chronic diseases, BMI, physical frailty, sleep disorders, and the aging attitude	9
Wei, 2024 (19)	China	①	Long-term care facilities	77.11 ± 9.65	179/169	OFI-6 score	31.03%	Age, education attainment, hypertension, visual impairment, leisure activities score, dietary diversity, ADL, cognitive impairment, depression	7
Yang, 2024 (24)	China	①	Community	70 (60–90)	149/158	OFI-8 score	21.17%	physical frailty, executive function	9
Yin, 2024 (26)	China	①	Community	70.00 ± 5.93	150/160	OFI-8 score	69.03%	sedentary time, passive smoking	9
Hiltunen, 2021 (44)	Finland	①	Long-term care facilities	82.00 ± 8.31	97/252	OF-checklist (six signs)	17.77%	Gender, BMI, MNA, diabetes, dementia, number of medications, MMSE score, eats independently, manages oral hygiene independently, physical frailty	7
Julkunen, 2024 (45)	Finland	①	Hospital	83.24 ± 8.57	82/221	OF-checklist (five signs)	53.00%	High oral disease burden, asymptomatic dental score	6
Ayoob, 2024 (47)	India	①	Health center	68.00 ± 6.02	140/110	OFI-6 score	67.00%	Physical frailty	6
Arai, 2024 (27)	Japan	①	Dental clinic	80.00 ± 4.40	860/1330	OFI-6 score	44.40%	Age, gender, frailty, medical expenses, medical consultation, dental expenses, dental consultation	8
Baba, 2021 (28)	Japan	①	Community	74.20 ± 6.10	58/152	OFI-6 score	8.10%	Age, physical frailty, and removable denture use	8
Hironaka, 2020 (29)	Japan	①	Community	73.30 ± 6.60	267/415	OFI-6 score	9.50%	Age, gender, education, MNA-SF score, MMSE score, depression, hypertension, heart disease, diabetes mellitus, hyperlipidemia, osteoporosis, osteoarthritis, spinal stenosis, malignant neoplasm, anemia, stroke, fracture, medications ≥5, social frailty, physical frailty	8
Hoshino D, 2020 (10)	Japan	①	Community	75.90 ± 6.30	198/283	OFI-6 score	21.20%	dietary variety score	8
Ishii, 2022 (30)	Japan	①	Internal medicine clinic	79.70 ± 3.80	72/39	OFI-8 score	53.20%	physical frailty	9
Iwasaki, 2020a (31)	Japan	①	Community	77.00 ± 4.80	428/626	OFI-6 score	20.40%	Age, annual income <3 million JPY, BMI, malnourishment, low physical activity, poor appetite, JST-IC score, depressive symptoms, polypharmacy, cognitive impairment	9
Iwasaki, 2020b (32)	Japan	③	Community	76.40 ± 4.10	191/275	OFI-6 score	14.40%	Denture use, annual income < 3 million JPY, low physical activity, poor appetite, JST-IC score, depressive symptoms	9
Iwasaki, 2021 (33)	Japan	①	Community	77.00 ± 4.70	439/643	OFI-6 score	20.98%	Age, educational status, annual income < 3 million JPY, denture use, smoking, low physical activity, vision impairment, chronic lower back pain, chronic knee pain	8
Iwasaki, 2024 (3)	Japan	①	Community	74.70 ± 5.50	626/580	OFI-5 score	36.70%	Age, years of education, low dietary variety, social isolation, physical frailty, drinking alcohol, cognitive impairment, and number of comorbidities	8
Izutsu, 2023 (34)	Japan	①	Community	76.60 ± 5.80	70/168	chewing function, swallowing function, and oral moisture	10.50%	diabetes, history of cancer, denture wearing, malnutrition	6
Kawamura, 2024 (35)	Japan	①	Hospital	77.20 ± 5.70	48/63	OFI-6 score	38.00%	Age, sarcopenia	8
Komatsu, 2021 (6)	Japan	①	Community	72.80 ± 5.50	116/264	OFI-6 score	14.00%	Age, cardiovascular disease, education, social activity, social activity, physical frailty	9
Kusunoki, 2023 (36)	Japan	①	Hospital	77.70 ± 6.60	128/123	OFI-8 score	38.65%	Gender, grip strength, Cr/CysC, eGFRcys/eGFRcre	5
Nakagawa, 2024 (37)	Japan	①	Dental clinics	79.90 ± 4.30	1094/1633	OFI-6 score	44.30%	Age, gender, BMI, frailty screening index, smoking history, years of education, number of medications taken a day, simplified nutritional appetite questionnaire, dietary variety score	8
Nishimoto, 2023 (38)	Japan	②	Community	72.20 ± 5.10	607/627	OFI-6 score	23.10%	Age, MMSE score, GDS-15 score, polypharmacy (≥6 medications), diabetes, osteoporosis, stroke	7*
Ohara, 2020 (39)	Japan	①	Community	79.10 ± 4.50	445/277	OFI-6 score	19.30%	Age, dietary variety score, eating alone, TMIG-IC, GDS-15 score	10
Tamaki, 2024 (43)	Japan	①	Dental clinics	80	1911/1311	OFI-8 score	37.78%	Remaining teeth, decayed teeth, oral malodor, family dental clinic, oral concerns	8
Tanaka, 2018 (40)	Japan	②	Community	73.00 ± 5.5	522/486	OFI-6 score	15.86%	Age, education level, early income (≤1.4 million yen), GDS-15 score, sarcopenia, physical frailty, number of prescribed medications	8*
Watanabe, 2024 (41)	Japan	②	Community	73.60 ± 6.00	5309/6065	OFI-8 score	46.91%	Age, smoking, drinking alcohol, HSES, medication, physical/psychological frailty	8*
Yamamoto, 2022 (42)	Japan	①	Dental clinics	≥65	385/458	OFI-6 score	23.87%	≥85 years Old, number of teeth present	8
Park HJ, 2024 (46)	Korea	①	Community	≥65	56/150	OFI-6 score	44.70%	Age	7

Study design: ① cross-sectional study; ② cohort study; ③ longitudinal study. NOS/AHRQ Score: *cohort studies. OF, oral frailty; NOS, Newcastle-Ottawa quality scale; AHRQ score, the agency for healthcare research and quality; OFI-6 score, oral frailty index-6; BMI, body mass index; MNA, mini nutritional assessment; MMSE, the mini-mental state examination; JST-IC, Japan Science and Technology Agency Index of Competence; SMI, skeletal muscle mass index; MNA-SF, Mini Nutritional Assessment-Short Form; OFI-8, oral frailty index-8; JPY, Japanese Yen; CNY, Chinese Yuan; OFI-5, oral frailty index-5; Cr, creatinine; CysC, cystatin C; eGFR, estimated glomerular filtration rate; OHAT, the oral health assessment tool; GSEOH, the geriatric self-efficacy scale for oral health; GOHAI, general oral health assessment index; TMIG-IC, Tokyo Metropolitan Institute of Gerontology Index of Competence; ADL, activities of daily living; GDS-15 score, the 15-item geriatric depression scale; TMIG-IC, Tokyo Metropolitan Institute of Gerontology Index of Competence; HSES, high socioeconomic status.

### Assessment of risk of bias

Risk of bias assessment was conducted independently by two researchers (Chen and Zhang) (11) was used to evaluate cross-sectional studies, and the Newcastle-Ottawa Quality Scale (NOS) (12) was used for cohort and case–control studies. The AHRQ cross-sectional study evaluation criteria consist of 11 items, each answered as “yes,” “no,” or “unclear.” Only “yes” scores 1, while “no” and “unclear” score 0. Studies with scores ranging from 8 to 11 were regarded as high quality, those with 4–7 as moderate quality, and those with 0–3 as low quality. The NOS includes 8 items with a total score of 9. Studies with a score greater than 6 were classified as high quality, those scoring 4–6 as moderate quality, and those scoring 0–3 as low quality. Any disagreements were resolved through discussions among three researchers (Chen, Zhang, and Yan).

### Statistical analysis

The statistical software used in this study for data analysis was RevMan5 (version 5.4) and Stata 16.0. Count data were summarized using odds ratios (ORs) with 95% confidence intervals (CIs), and continuous data were expressed as weighted mean differences (WMDs) with 95% CIs. For continuous data assessed using different measurement tools or units across the included studies, standardized mean differences (SMD) were used. Heterogeneity across all included studies was assessed and quantified using Cochrane Q and *I^2^* statistics, respectively. *I^2^* ≥ 50% indicated that heterogeneity across included studies was significant; therefore, a random-effect model was subsequently used to pool these results. A fixed-effects model was used when heterogeneity was not significant (*I^2^* < 50%). A *p*-value of < 0.05 was considered statistically significant. Sensitivity analysis was performed by eliminating each study individually to assess the consistency and quality of the results. Funnel plots and Egger’s test were used to assess publication bias for influencing factors of reported by≥10 studies, with a *p*-value of >0.05, indicating that there was no significant publication bias.

## Results

### Study selection

**Figure 1:**
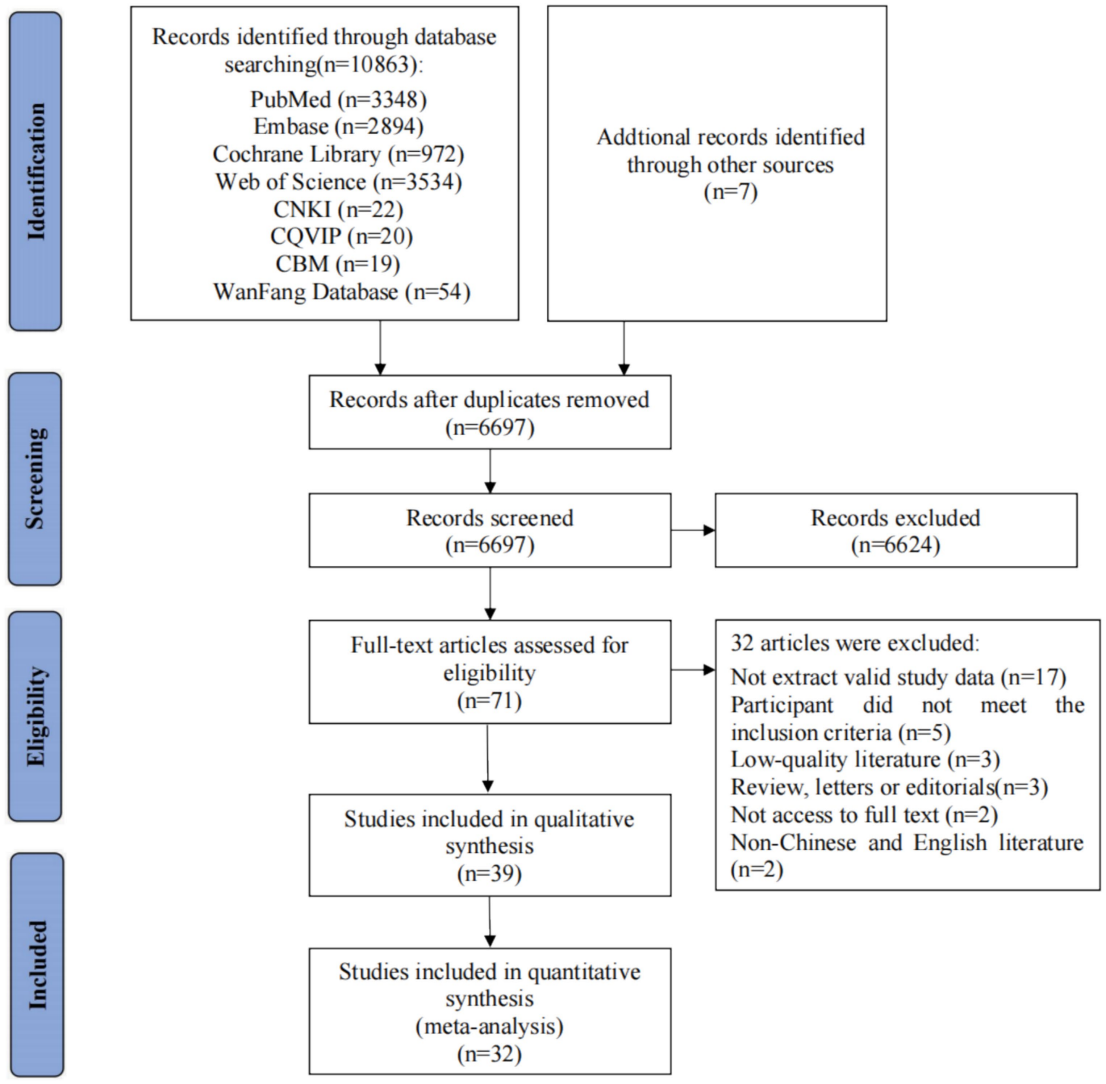
Flow chart of the process of selecting eligible studies.

A total of 10,863 records were identified after database searches. In addition, seven articles were supplemented by consulting the included references. A total of 6,697 studies remained after removing duplicates. Finally, 71 records were required for full-text assessments. The reasons for excluding records were as follows: (1) not extracting valid study data (*n* = 17); (2) participants who did not meet the inclusion criteria (*n* = 5); (3) low-quality literature (*n* = 3); (4) reviews, letters, or editorials (*n* = 3); (5) no access to full text (*n* = 2); and (6) non-Chinese and English literature (*n* = 2). Finally, 39 studies, including 35 cross-sectional studies, 3 cohort studies, and 1 longitudinal study, were selected for this systematic review. A total of 32 of the 39 studies were included in the meta-analysis (Figure 1).

### Characteristics of the included studies and quality assessment results

Table 1 shows the selected studies that reported factors associated with OF in older adults. The included studies will be published between 2018 and 2025. The sample size for each study ranged from 111 to 11,374, and 41,854 older adults were included in this review. The majority of the studies were conducted in China (5, 13–26) (*n* = 15) and Japan (3, 6, 10, 27–43) (*n* = 20), 2 in Finland (44, 45), 1 in Korea (46), and 1 in India (47). The prevalence of among older adults ranged from 8.10 to 71.55%. The included studies used a variety of oral frailty assessment tools, predominantly the oral frailty assessment scale. Among these, the OFI-6 score was used in 18 studies (6, 10, 19, 25, 27–29, 31–33, 35, 37–40, 42, 46, 47), whereas the OFI-8 score was employed in 17 studies (5, 13–18, 20–24, 26, 30, 36, 41, 43), making it the most frequently used assessment tool in the literature. Additionally, one study incorporated the OFI-5 score (3). All included cohort studies were of high quality, and the quality of the cross-sectional studies was moderate or above. The basic characteristics of the included studies and the results of the literature evaluation are shown in Table 1.

OF checklist (six signs): dry mouth, food residue on oral surfaces, unclear speech, inability to keep the mouth open, pain during clinical oral examination, and a pureed/soft diet. OF checklist (five signs): having a diet of soft/pureed food, residue of food in the oral cavity, inability to keep the mouth open during examination, unclearness of speech, and dry mouth.

### Factors associated with OF in older adults

Table 2 shows the meta-analysis results of factors associated with OF in older adults that were identified in two or more studies. We extracted 27 factors, and the results showed that 20 factors were significantly different between the OF and non-OF groups (*p* < 0.05). All these factors were further categorized into sociodemographic, comorbidity, physical function, social, psychological, and dietary factors.

**Table 2:** Meta-analyses of the influence factors of in older frailty.

No.	Influence factors	Number of studies	Estimation of combined			Effect model	Heterogeneity test	
			OR/WMD (95% CI)	Z	*P*		I^***2***^	*P*
Sociodemographic Factors								
1	Age^*^	15 (3, 6, 13, 19, 22, 27, 29, 31, 33, 35, 37–41)	4.21 (3.09–5.33)	7.36	<0.001	Random	98%	<0.001
	Age ≥ 80 years	7 (14, 16, 18, 21, 23, 24)	4.62 (1.90–11.21)	3.38	<0.001	Random	94%	<0.001
	Age ≥ 75 years	3 (20, 25, 42)	1.99 (1.55–2.57)	3.53	<0.001	Fixed	43%	0.170
2	Female	8 (16, 22, 23, 27, 29, 36, 37, 44)	1.76 (1.41–2.20)	5.02	<0.001	Random	73%	<0.001
3	years of education^*^	6 (3, 6, 29, 33, 37, 40)	0.68 (−0.04–1.41)	1.84	0.007	Random	96%	<0.001
	education level below junior high	4 (13, 17, 19, 22)	1.25 (0.56–2.78)	0.54	0.59	Random	92%	<0.001
4	Low income	7 (16, 17, 23, 31–33, 40)	1.94 (1.20–2.68)	3.98	<0.001	Random	82%	<0.001
5	BMI^*^	3 (31, 37, 44)	1.50 (0.41–5.59)	2.69	0.007	Random	92%	<0.001
6	Drinks alcohol	5 (3, 16, 17, 20, 41)	1.25 (0.58–2.69)	0.57	0.570	Random	98%	<0.001
7	Smoking	6 (22, 23, 26, 33, 37, 41)	1.20 (0.88–1.63)	1.14	0.250	Random	89%	<0.001
Comorbidity Factors								
8	Diabetes mellitus	5 (17, 18, 29, 34, 38)	1.56 (0.95–2.57)	1.76	0.080	Random	76%	0.002
9	Hypertension	2 (19, 29)	1.18 (0.53–2.60)	0.40	0.690	Random	63%	0.100
10	Sarcopenia	2 (18, 40)	2.92 (2.54–3.36)	15.09	<0.001	Fixed	0%	0.720
11	Stroke	2 (29, 38)	2.68 (1.28–5.61)	2.62	0.009	Random	63%	0.100
12	Osteoporosis	2 (29, 38)	1.50 (1.05–2.13)	2.25	0.020	Fixed	0%	0.590
13	Malignant neoplasm	2 (29, 34)	1.50 (0.22–10.42)	0.41	0.680	Random	86%	0.007
14	Cognitive impairment	3 (3, 19, 31)	4.25 (1.72–10.51)	3.13	0.002	Random	80%	0.007
	Cognition status MMSE	3 (29, 38, 44)	1.55 (0.30–2.81)	2.43	0.020	Random	93%	<0.001
15	≥2 chronic conditions	4 (14, 16, 20, 22)	6.07 (2.32–15.90)	3.67	<0.001	Random	90%	<0.001
16	Taking ≥ 5 medications	5 (17, 22, 29, 31, 38)	2.48 (1.59–3.88)	4.01	<0.001	Random	81%	<0.001
17	Poor sleep quality	3 (16, 17, 21)	3.79 (1.47–9.80)	2.75	<0.001	Random	95%	<0.001
Physical Function Factors								
18	Physical frailty	11 (3, 6, 14, 17, 22, 24, 29, 30, 40, 44, 47)	2.21 (1.63–3.00)	5.073	<0.001	Random	87%	<0.001
19	Low physical activity level	3 (31–33)	1.77 (1.41–2.22)	4.91	<0.001	Fixed	0%	0.580
20	ADL (impairment)	2 (19, 20)	4.46 (1.81–11.01)	3.24	0.001	Random	80%	0.020
Social and Psychological Factors								
21	Depressive symptoms	4 (19, 25, 29, 32)	3.76 (2.69–5.24)	7.80	<0.001	Fixed	0%	0.450
	Depression (GDS-15 score)	3 (38–40)	0.40 (0.16–0.63)	3.29	0.001	Random	88%	<0.001
22	Social isolation	2 (3, 18)	1.95 (1.43–2.65)	4.25	<0.001	Random	77%	0.04
Dietary Factors								
23	Malnutrition	3 (17, 20, 34)	2.58 (1.55–4.28)	3.67	<0.001	Random	59%	0.080
24	Dietary variety score	3 (10, 37, 39)	1.35 (0.56–3.26)	0.66	0.510	Random	80%	0.007
25	Low dietary variety	2 (3, 10)	1.69 (1.37–2.08)	4.89	<0.001	Fixed	0%	0.990
26	Poor appetite	2 (31, 32)	2.11 (1.62–2.74)	5.55	<0.001	Fixed	0%	1.000
27	Eating alone	2 (39, 44)	0.51 (0.05–5.16)	0.57	0.570	Random	97%	<0.001

#### Sociodemographic factors

Seven sociodemographic factors were assessed in our review: age, gender, education, low income, BMI, smoking, and alcohol consumption. The results of the meta-analysis showed that age (WMD = 4.21, 95% CI: 3.09–5.33), age ≥ 80 years (OR = 4.62, 95% CI: 1.90–11.21), age ≥ 75 years (OR = 1.99, 95% CI: 1.55–2.57), female sex (OR = 1.76, 95% CI: 1.41–2.20), years of education (WMD = 0.68, 95% CI: −0.04-1.41), low income (OR = 1.94, 95% CI: 1.20–2.68), and BMI (WMD = 1.50, 95% CI: 0.41–5.59) were associated with OF in older adults (*p* < 0.05). There was no association between education level below junior high, smoking, or drinking alcohol with OF in older adults.

#### Comorbidity factors

A total of 10 comorbidity factors were identified: diabetes mellitus, hypertension, sarcopenia, stroke, osteoporosis, malignant neoplasm, cognitive impairment, ≥2 chronic conditions, taking ≥ 5 medications, and poor sleep quality. Among them, sarcopenia (OR = 2.92, 95% CI: 2.54–3.36), stroke (OR = 2.68, 95% CI:1.28–5.61), osteoporosis (OR = 1.50, 95% CI: 1.05–2.13), cognitive impairment (OR = 4.25, 95% CI:1.72–10.51), cognition status MMSE (WMD = 1.55, 95% CI: 0.30–2.81),≥2 chronic conditions (OR = 6.07, 95% CI:2.32–15.90), taking ≥ 5 medications (OR = 2.48, 95% CI:1.59–3.88), and poor sleep quality (OR = 3.79, 95% CI:1.47–9.80) were associated with OF in older adults (*p* < 0.05). There was no association between OF and diabetes mellitus, hypertension, or malignant neoplasms in older adults.

#### Physical function factors

Physical function factors, including physical frailty (OR = 2.21, 95% CI: 1.63–3.00), low physical activity level (OR = 1.77, 95% CI: 1.41–2.22), and ADL impairment (OR = 4.46, 95% CI: 1.81–11.01), were all found to be significantly associated with OF in older adults.

#### Social and psychological factors

Social and psychological factors, including depressive symptoms (OR = 3.76, 95% CI: 2.69–5.24), GDS-15 score (WMD = 0.40, 95% CI: 0.16–0.63), and social isolation (OR = 1.95, 95% CI:1.43–2.65), were all found to have a significant association with OF.

#### Dietary factors

Dietary factors, including malnutrition (OR = 2.58, 95% CI:1.55–4.28), low dietary variety (OR = 1.69, 95% CI:1.37–2.08), and poor appetite (OR = 2.11, 95% CI:1.62–2.74), were significantly associated with OF. No significant relationship was found between eating alone, dietary variety score, and OF.

### Subgroup analysis

The meta-results were stratified into two subgroups based on the oral frailty (OF) assessment tools used: OF-6 and OF-8 scores. In the OF-6 score subgroup, the following factors showed statistically significant differences between the OF and non-OF groups: age, female sex, low income, BMI, stroke, osteoporosis, cognitive impairment, taking ≥ 5 medications, physical frailty, low physical activity level, depressive symptoms, and poor appetite. In the OF-8 score subgroup, significant factors included age, female sex, low income, ≥ 2 chronic conditions, poor sleep quality, physical frailty, and malnutrition. Common influencing factors identified in both subgroups were age, female sex, low income, and physical frailty (all *p* < 0.05). The detailed results of the subgroup analysis are provided in Supplementary Table S2.

### Sensitivity analysis and publication bias

Sensitivity analysis was performed by eliminating each study individually, and the remaining articles were recombined for the meta-analysis. The results remained consistent after eliminating any individual study, indicating that the meta-analysis results were relatively stable. Funnel plots were employed to independently evaluate age and physical frailty, both of which have been recognized as significant determinants in at least 10 published studies. The results indicated that the funnel plots of physical frailty exhibited a consistent and symmetrical distribution, whereas the distribution of age was lopsided. To identify the source of asymmetry, we conducted a sensitivity analysis by sequentially excluding studies. The asymmetry was largely resolved upon exclusion of the study by Tu et al., which indicated that it was the primary contributor. This outlier effect may be attributed to its relatively small sample size (*n* = 204) compared to the other included studies. Although Egger’s test did not show a significant publication bias for age (*p* = 0.231) or physical frailty (*p* = 0.946), the initial visual asymmetry for age suggests that potential bias cannot be fully excluded, and the corresponding results should be interpreted with caution.

## Discussion

In the context of the rapidly increasing global aging population, geriatric oral health has become a significant public health issue (4). Notably, oral frailty, which is characterized as a state between robust oral function and its decline (40), has received increasing attention and has become one of the research hotspots in the field of geriatrics. In this review, we included 39 studies involving 41,854 older adults (> 60 years). These studies informed our analysis, which showed that the global prevalence of oral fragility in older adults was 34.1% (29.1–39.2%). As a developing country with a large older population, China (41.2%) has a substantially higher prevalence of oral frailty than Japan (27.0%), which is consistent with earlier research. This illustrates that the oral health status of older adults is concerning, and there is an urgent need for early risk screening and focused oral health management.

This study was a comprehensive systematic meta-analysis of factors associated with OF in older adults. We found that OF was significantly associated with 20 factors across 5 categories in older adults: sociodemographic factors including advanced age, female sex, years of education, low income, and BMI; comorbidity factors including sarcopenia, stroke, osteoporosis, cognitive impairment, ≥2 chronic conditions, taking ≥ 5 medications, and poor sleep quality; physical function factors including physical frailty, low physical activity level, and ADL impairment; social and psychological factors including depression and social isolation; and dietary factors including malnutrition, low dietary variety, and poor appetite.

### Sociodemographic factors

Our meta-analysis revealed that advanced age is a risk factor for OF in older adults, regardless of whether continuous variables were considered or whether the groups aged 80 (14, 16, 18, 21, 23, 24, 26) and above and those aged 75 (20, 25, 42) and above were analyzed. Previous studies have shown that as age increases, the activity of alkaline phosphatase in periodontal ligament cells, along with their regenerative and osteogenic capabilities, declines. Additionally, older adults experience physiological changes such as gingival atrophy, demineralization, and softening of cementum, which can lead to conditions such as periodontitis and dental caries (5). The prevalence of among older females is higher than that observed in their male counterparts. This difference may be explained by the earlier development of permanent dentition in females, which leads to a prolonged period of exposure to the effects of masticatory wear and bacterial degradation (22). Moreover, postmenopausal older women experience diminished estrogen levels, which contribute to an increased loss of bone calcium. This process is subsequently associated with alveolar bone atrophy, reduced salivary secretion, decreased salivary flow rate, and heightened vascular permeability. These physiological alterations heighten susceptibility to various oral health complications, including xerostomia, dental caries, and periodontal disease (48). Therefore, special attention ought to be directed to oral health among older adults, particularly older women.

The results of this meta-analysis showed that years of education were risk factors for OF in older adults; those with advanced age and lower educational attainment often have limited oral health knowledge and literacy, which in turn leads to suboptimal oral health behaviors and poorer outcomes (49). Moreover, older individuals with limited income may refrain from seeking dental examinations or purchasing oral hygiene products because of cost concerns. Over time, this avoidance can progressively worsen oral health conditions (50). Consequently, in managing OF among older adults, health administrators should prioritize this vulnerable group and develop simple, cost-effective strategies tailored to their needs to enhance intervention efficacy.

In addition, our meta-analysis demonstrated that BMI is a significant risk factor for OF in older adults, with those with OF exhibiting a significantly lower BMI. As an objective biomarker of nutritional status, low BMI in older adults strongly correlates with malnutrition risk and physical frailty progression (30). These findings underscore the necessity for healthcare providers to implement targeted nutritional interventions and comprehensive frailty assessments as integral components of oral frailty management strategies in at-risk older adults.

### Comorbidity factors

Our findings indicate that sarcopenia is an independent factor associated with OF in older adults, which is consistent with prior evidence (40). The progression of in older adults may increase the risk of sarcopenia, as confirmed by various studies. Conversely, sarcopenia may contribute to diminished masticatory muscle strength and reduced swallowing reflex sensitivity in older adults. This increases the probability of chewing and swallowing difficulties, which can restrict dietary options for older adults. Over time, this may exacerbate oral dysfunction, leading to insufficient nutritional intake, prolonged meal duration, and increased eating discomfort (18). Similarly, osteoporosis, which is prevalent among older adults, has been identified as having an independent association with OF. Older patients with osteoporosis often experience issues such as tooth loss and periodontal disease, which may lead to oral dysfunction, such as chewing and swallowing difficulties.

In our meta-analysis, we found a significant association between cognitive impairment and OF in older adults. One potential explanation for this relationship is that declining oral health, including tooth loss and masticatory dysfunction, diminishes sensorimotor stimulation from chewing, which is vital for brain function (51). Conversely, older adults with cognitive impairment often lack awareness and the ability to maintain proper oral hygiene. This inadequacy increases the oral bacterial load and elevates inflammatory responses, heightening the risk of dental caries and tooth loss and subsequently increasing the likelihood of (52). Additionally, it is hypothesized that oral microbiota or inflammatory mediators may penetrate the central nervous system through circulatory or neural pathways, thereby intensifying the risk of cognitive disorders, including Alzheimer’s disease (53).

Similarly, a significant association was observed between OF and stroke. First, swallowing and chewing difficulties common in stroke patients can cause saliva and food to stagnate in the oral cavity, subsequently impairing oral function. Second, stroke-related cognitive and motor impairments reduce older adults’ ability to maintain oral hygiene, which exacerbates their oral health status, potentially leading to the onset of oral diseases or worsening of pre-existing conditions, and may even result in tooth loss (54). Conversely, chronic infection by periodontal pathogens and the associated immune response have been identified as factors that can elevate the risk of stroke, establishing them as independent risk factors for ischemic stroke (55). Furthermore, this review found that older adults with poor sleep quality had a 3.79-fold higher risk of developing OF than those with normal sleep quality. Hu (5) and Tang (21) noted that given the well-established association between sleep quality and physical frailty, reduced sleep quality in older adults is linked to greater PF severity, which in turn impacts their oral health.

≥2 chronic conditions and polypharmacy have also been identified as significant risk factors for OF in older adults. The prevalence of polypharmacy in older adults often stems from multiple comorbidities. A meta-analysis of studies conducted in China revealed that 40.2% of adults aged ≥80 years exhibited at least one concomitant condition (56). Long-term polypharmacy in the context of chronic diseases often affects saliva secretion and flow rate, leading to xerostomia (40). This reduces oral self-cleaning capacity, elevating the risk of caries, periodontitis, oral candidiasis, impaired masticatory function, and dysphagia (5). Meanwhile, the accumulation of adverse events related to comorbidities and polypharmacy is associated with both general health deterioration and oral function decline. Consequently, it is imperative that clinical healthcare providers closely monitor the oral health of older patients presenting with comorbidities and polypharmacy. Regular oral assessments should be performed, accompanied by appropriate oral health education initiatives. Therefore, relevant complications and oral diseases should be actively treated. Simultaneously, measures must be taken to ensure that older individuals utilize medications in a safe, accurate, and judicious manner to mitigate their negative impacts on oral health.

### Physical function factors

The association between physical frailty and OF may be attributed to a decline in physiological reserve function, physical activity, and physical strength. This decline leads to diminished opportunities for oral communication and reduced mobility of the oral and maxillofacial muscles, including the tongue, which ultimately results in impaired chewing and swallowing abilities (5). In addition, this decline impairs their ability to maintain proper oral hygiene, such as brushing and rinsing, ultimately leading to suboptimal oral health (14).

Physical activity, a subdomain of physical frailty, tends to decline with age in older adults. As previous studies have shown, reduced masticatory ability is significantly correlated with low physical activity levels and slow gait speed in older adults (57). Likewise, impairments in activities of daily living are significantly associated with OF in older adults. One possible explanation is that greater physical frailty may hinder older adults’ ability to perform daily activities; conversely, the ability to engage in activities of daily living can also influence oral hygiene practices (58). The above results suggest that clinical healthcare providers should assist frail older adult patients in enhancing their physical function through exercise, as this intervention may contribute to a decreased risk of developing oral frailty.

### Social and psychological factors

We found that social and psychological factors, including depression and social isolation, are significantly associated with OF in older adults. Social isolation is defined as the objective state of having few social relationships and/or infrequent social contact with others (59). There is evidence that socially isolated older adults were more vulnerable to functional disability, which may lead to infrequent oral hygiene behaviors and a higher risk of systemic inflammation, ultimately resulting in tooth loss (60). Oral microorganisms play an important role in the relationship between depression and oral health. First, high concentrations of cortisol in patients with depression can be secreted into the mouth through the salivary glands, which has an impact on oral health and the oral microbiome, increasing the risk of dental caries, periodontal disease, and oral dryness syndrome (61). On the other hand, oral microbes and their metabolites can enter the intestine and migrate to the damaged blood–brain barrier, leading to neuroinflammation and promoting the development of depression (62). Consequently, when developing OF management strategies, oral healthcare providers must consider the psychosocial status of older adults while emphasizing the importance of their social support networks. Encouraging older adults to participate in community activities can alleviate OF by enhancing social support and promoting health-related behaviors (63).

### Dietary factors

Dietary status is closely related to oral health in older adults. The results of this meta-analysis showed that low dietary variety and poor appetite were significantly associated with OF in older adults. Previous studies have found that food intake type can affect older adults’ oral health. For example, reduced intake of milk and green leafy vegetables increases the risk of periodontal disease and tooth loss (10). Nakagawa et al. (37) found that there may be a detrimental cycle between low appetite and OF; in older adults, a compromised ability to consume specific foods due to deteriorating oral function might lead to poor appetite. Poor appetite, in turn, limits dietary variety and aggravates OF. This suggests that assessing dietary variety and appetite may help understand the risk of in older adults. In addition, our review found an association between OF and malnutrition. A probable cause is that older adults with compromised masticatory function frequently steer clear of difficult-to-eat foods, including meat, fruits, and vegetables, and these dietary habits increase their vulnerability to malnutrition (30). Furthermore, the susceptibility of type II muscle fibers, which play a critical role in the swallowing process, to nutritional deficiencies may elucidate the mechanism by which undernutrition can lead to or exacerbate OF (16). These findings further underscore the significance of dietary and nutritional evaluations in the management of oral frailty in older adults.

### Comparison of influencing factors across different assessment tools

Given the variations in OF assessment tools across the included studies, we performed a subgroup analysis. The OF-8 and OF-6 scores were the two most frequently used OF assessment tools in this study. We conducted separate meta-analyses for each group to explore the respective risk factors. A total of 18 studies used OF-6, whereas 17 used OF-8. The results showed that the different tools revealed partly distinct influencing factors. Age, female sex, low income, and physical frailty emerged as common factors in both subgroups, confirming their key role in predicting oral frailty among older adults. These findings also underscore the importance of using uniform assessment tools in future research to enhance the consistency and comparability of the results.

### Limitations

The present study has the following limitations: First, most of the literature included in this review was cross-sectional, which inherently limits the ability to establish causal relationships. Given that OF is a dynamic and reversible condition, there is an urgent need for high-quality future longitudinal studies to elucidate the causal association between various factors and OF in older adults. Second, most research has been conducted in East Asia (particularly Japan and China), which may restrict its applicability to other demographic groups. Third, the meta-analysis exhibited significant heterogeneity, potentially due to regional differences, variations in demographic characteristics, sample sizes, and study designs, as well as inconsistent OF assessment tools. Future studies should prioritize large-scale, multicenter research with standardized assessment tools for OF to enhance data credibility and comparability. Finally, the association results for certain factors with OF showed wide confidence intervals, reflecting limited precision. This likely stems from the small number of primary studies on these factors, coupled with variations in sample sizes and measurement tools. These findings necessitate cautious interpretation and highlight the need for further research to validate these associations.

## Conclusion

In summary, this systematic review found that OF in older adults is associated with advanced age, female sex, years of education, low income, BMI, sarcopenia, stroke, osteoporosis, cognitive impairment, ≥2 chronic conditions, polypharmacy, poor sleep quality, physical frailty, low physical activity level, ADL impairment, depression, social isolation, malnutrition, low dietary variety, and poor appetite. It is recommended that public health policymakers and healthcare professionals conduct a thorough assessment of the associated factors and establish early screening initiatives as well as implement effective preventive and intervention strategies to mitigate oral frailty among the older population.

## Data Availability

The original contributions presented in the study are included in the article/Supplementary material, and further inquiries can be directed to the corresponding author.
