# Supplementary material for: Factors associated with oral frailty in older adults: a systematic review and meta-analysis
Source: Front Public Health. 2025 Dec 10;13:1688322. doi: 10.3389/fpubh.2025.1688322 (PMC12727602; doi:10.3389/fpubh.2025.1688322)
Supplement: Supplementary file 1 [file Table_1.DOCX]

**Table S1**| Search strategy of PubMed.

| **#** | **Search** |
| --- | --- |
| #1 | aged[MeSH Terms] |
| #2 | aged[tiab] OR elderly[tiab] OR senior citizen[tiab] OR older adult[tiab] OR seniors[tiab] OR old people[tiab] OR advanced age[tiab] OR older individuals[tiab] |
| #3 | #1 OR #2 |
| #4 | oral frail*[tiab] OR oral weakness[tiab] OR oral health[tiab] |
| #5 | risk factor[tiab] OR root cause analysis[tiab] OR causalit*[tiab] OR influencing factors[tiab] OR affecting factors[tiab] OR dangerous factors[tiab] OR relevant factors[tiab] OR relat*[tiab] OR correlat*[tiab] OR associat*[tiab] |
| #6 | #3 AND #4 AND #5 |
