# Supplementary material for: Factors associated with oral frailty in older adults: a systematic review and meta-analysis
Source: Front Public Health. 2025 Dec 10;13:1688322. doi: 10.3389/fpubh.2025.1688322 (PMC12727602; doi:10.3389/fpubh.2025.1688322)
Supplement: Supplementary file 2 [file Table_2.DOCX]

| **Assessment tool** | **Influence factors** | **Number of studies** | **Estimation of combined** | | | **Effect model** | **Heterogeneity test** | |
| --- | --- | --- | --- | --- | --- | --- | --- | --- |
|  |  |  | ***OR/WMD(95% CI)*** | ***Z*** | ***P*** |  | ***I^2^*** | ***P*** |
| OFI-6 Score |  |  |  |  |  |  |  |  |
|  | Age^*^ | 11 | 3.79 (2.67-4.92) | 6.61 | <0.001 | Random | 96% | <0.001 |
|  | Female | 3 | 1.30 (1.16-1.45) | 4.50 | <0.001 | Fixed | 0% | 0.86 |
|  | Low income | 4 | 1.43 (1.22-1.68) | 4.42 | <0.001 | Fixed | 0% | 0.79 |
|  | BMI^*^ | 2 | 2.00 (1.78-2.22) | 18.19 | <0.001 | Fixed | 0% | 0.99 |
|  | Stroke | 2 | 2.68 (1.28-5.61) | 2.62 | 0.009 | Random | 63% | 0.100 |
|  | Osteoporosis | 2 | 1.50 (1.05-2.13) | 2.25 | 0.020 | Fixed | 0% | 0.590 |
|  | Cognitive impairment | 2 | 6.95 (4.30-11.23) | 7.93 | <0.001 | Fixed | 0% | 0.71 |
|  | Types of medications ≥ 5 | 3 | 1.81 (1.46-2.24) | 5.47 | <0.001 | Fixed | 0% | 0.65 |
|  | Physical frailty | 4 | 3.63 (2;73-4.83) | 8.90 | <0.001 | Fixed | 0% | 0.78 |
|  | Low physical activity level | 3 | 1.77 (1.41-2.22) | 4.91 | <0.001 | Fixed | 0% | 0.580 |
|  | Depression symptoms | 4 | 3.76 (2.69-5.24) | 7.80 | <0.001 | Fixed | 0% | 0.450 |
|  | Poor appetite | 2 | 2.11 (1.62-2.74) | 5.55 | <0.001 | Fixed | 0% | 1.000 |
| OFI-8 Score |  |  |  |  |  |  |  |  |
|  | Age^*^ | 3 | 6.69 (1.79-11.60) | 2.68 | 0.007 | Random | 99% | <0.001 |
|  | Female | 4 | 2.34 (1.61-3.40) | 4.48 | <0.001 | Random | 68% | 0.02 |
|  | Low income | 3 | 2.90 (1.39-6.08) | 2.83 | 0.005 | Random | 89% | <0.001 |
|  | ≥2 chronic conditions | 4 | 6.07 (2.32-15.90) | 3.67 | <0.001 | Random | 90% | <0.001 |
|  | Poor sleep quality | 3 | 3.79 (1.47-9.80) | 2.75 | <0.001 | Random | 95% | <0.001 |
|  | Physical frailty | 6 | 7.23 (1.81-28.87) | 2.80 | <0.001 | Random | 97% | <0.001 |
|  | Malnutrition | 2 | 2.85 (1.39-5.84) | 2.87 | 0.004 | Random | 79% | 0.030 |

Table S2. Subgroup analysis of the influencing factors of OF using assessment tools.
